# Smart Lattice Structures with Self-Sensing Functionalities via Hybrid Additive Manufacturing Technology

**DOI:** 10.3390/mi15010002

**Published:** 2023-12-19

**Authors:** Liu He, Peiren Wang, Junhui Yang, Kaoyi Fan, Hanqiang Zhang, Luyan Zhang, Mingxing Jiang, Xiaoyi Chen, Zhen Chen, Min Chen, Haiyun Liu, Ji Li

**Affiliations:** 1 Key Laboratory of MEMS of the Ministry of Education, Southeast University, Nanjing 210096, China; 220211687@seu.edu.cn (L.H.); wang_peiren@seu.edu.cn (P.W.); 220221728@seu.edu.cn (J.Y.); 220216137@seu.edu.cn (K.F.); hanqiang_zhang@seu.edu.cn (H.Z.); 220216021@seu.edu.cn (L.Z.); 220226180@seu.edu.cn (M.J.); 220225906@seu.edu.cn (X.C.); 220226165@seu.edu.cn (Z.C.); 2 School of Advanced Technology, Xi’an Jiaotong-Liverpool University, Suzhou 215123, China; min.chen@xjtlu.edu.cn; 3 College of Computer and Information, Hohai University, Nanjing 211100, China; haiyun_liu@hhu.edu.cn

**Keywords:** additive manufacturing, electroless plating, lattice structures, self-sensing

## Abstract

Lattice structures are a group of cellular materials composed of regular repeating unit cells. Due to their extraordinary mechanical properties, such as specific mechanical strength, ultra-low density, negative Poisson’s ratio, etc., lattice structures have been widely applied in the fields of aviation and aerospace, medical devices, architecture, and automobiles. Hybrid additive manufacturing (HAM), an integrated manufacturing technology of 3D printing processes and other complementary processes, is becoming a competent candidate for conveniently delivering lattice structures with multifunctionalities, not just mechanical aspects. This work proposes a HAM technology that combines vat photopolymerization (VPP) and electroless plating process to fabricate smart metal-coated lattice structures. VPP 3D printing process is applied to create a highly precise polymer lattice structure, and thereafter electroless plating is conducted to deposit a thin layer of metal, which could be used as a resistive sensor for monitoring the mechanical loading on the structure. Ni-P layer and copper layer were successfully obtained with the resistivity of $8.2\times 10^{-7}\Omega \cdot m$ and $2.0\;\times 10^{-8}\;\Omega \cdot m$ , respectively. Smart lattice structures with force-loading self-sensing functionality are fabricated to prove the feasibility of this HAM technology for fabricating multifunctional polymer-metal lattice composites.

## 1. Introduction

The lattice structure is a kind of cellular material with the regular repeating structure of their unit cell [1]. Owing to its extraordinary mechanical performances, e.g., specific mechanical strength [2], ultra-low density [3], tunable thermal conductivity [4], and negative Poisson’s ratio [5], etc., lattice structures have been applied to medical [6,7], energy absorption [8,9], aerospace [10,11], nuclear engineering [3], flexible and wearable devices [12]. Traditionally, lattice structures are fabricated via water jet cutting [13], investment casting [14], and wire-woven methods [15], which cause waste of materials, limited design flexibility, and difficulty in manufacturing microstructures.

Unlike traditional subtractive manufacturing methods, additive manufacturing (AM) technology creates 3D objects layer by layer based on computer-aided design (CAD) files, which offers high freedom of design and appropriate processing methods for the manufacture of lattice products. Nowadays, AM has been widely applied in the manufacturing of lattice structures. Typical examples include selective laser melting (SLM) technology, which was used to fabricate lattices with variable attitude octahedral structure and body-centered cuboctahedral structure [16]. Free-hanging fused deposition modeling (FDM) technique and free-hanging 3D printing method were developed for fabricating smart CCF (continuous carbon fiber)-thermoplastic lattice truss sandwich structures [17]. The lattice structure design enables customization of the mechanical properties of the material [18]. For practical applications such as car crash boxes and helmets [19,20], the working conditions of lattice parts are often severe, which poses a great challenge to the health of these parts. Therefore, the health conditions of the lattice parts need to be monitored. However, traditional AM can only deliver lattice structures with a single material and single process. These products normally provide only mechanical functionalities. In order to monitor the working conditions of lattice parts, integrated sensors should be made together with the lattice frame. For example, Abyzova et al. [21] prepared conductive wristbands for wearable smartwatches by combining AM and laser processing methods, expanding the possibilities of rGO/polymer composites for applications in flexible electronics. Therefore, diverse materials and processes are required to realize the integrated manufacturing of lattice structures and self-sensing functionalities.

Hybrid additive manufacturing (HAM) technology is a great advance for AM technology viand works by combining AM processes with other complementary processes [22]. For instance, by combining digital light processing (DLP) and vacuum casting processes, Deng et al. produced a reconfigurable lattice hand based on liquid metal lattice materials with shape memory functionality [23]. Yin et al. fabricated a pressure sensor with a programmable lattice structure via hybridizing DLP 3D printing and carbon nanotube (CNT) ultrasonication coating [24]. Kamat et al. used stereolithography (SLA) technology and the dip coating method to fabricate a piezoresistive pressure sensor with graphene nanoplatelets on the surface [25]. For the fabrication of 3D electronics, electroless plating (ELP) processes are also a preferable complementary technique in the HAM area; these processes can deposit various metallic and alloy coatings such as copper, silver, gold, nickel-phosphorus alloy, etc. on 3D substrate [26,27,28]. For example, Hensleigh et al. employed SLA printing to fabricate the dual-material structure with positive, negative, and neutral resins and soaked it in either a positive or negative catalyst solution to define the ELP deposition area for 3D electronics [29]. Shin et al. [30] used DLP and ELP techniques to deposit metal layers on the micro-lattice and the compressive stiffness of the lattice increased from 8.8 MPa to 11.1 MPa. Our group combined laser activated ELP with various AM technologies for the fabrication of high-resolution 3D conformal/embedded circuit boards, sensors, antennas, etc. [31,32,33,34,35].

**Figure 1:**
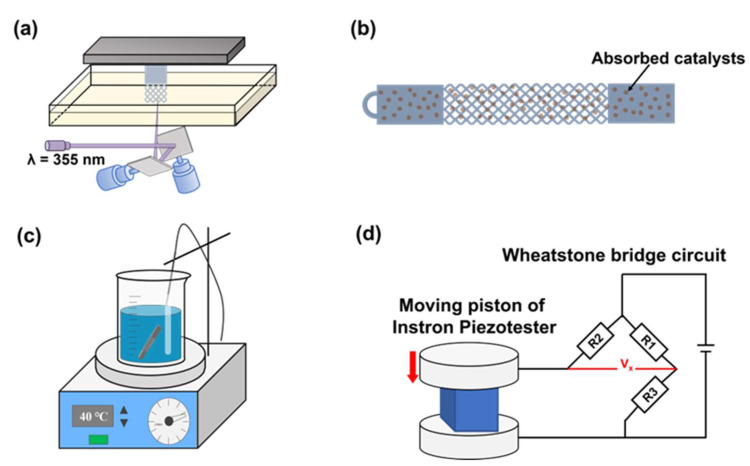
The fabrication process of the proposed HAM technology. (**a**) Stereolithography 3D printing. (**b**) Surface treatment and activation. (**c**) Electroless plating. (**d**) Schematic representation of the setup used for the smart lattice pressure sensor.

In this work, we proposed a HAM technology that integrated VPP and ELP process to fabricate smart lattice structures with force-loading self-sensing functionality (including both tensile and compressive loadings), which could not only exploit the mechanical advantages of lattice structure but also the self-sensing functionality. In this way, the health situation of the lattice structures could be monitored and evaluated in real-time. As shown in Figure 1, the lattice frameworks are fabricated by a high-precision SLA 3D printing process. After specific pretreatment steps, a nickel-phosphorus (Ni-P) film or copper film is electroless plated on the surface of a photopolymer lattice framework. The polymer-metal lattice composite possesses a self-sensing ability and therefore can be regarded as a smart structure. To fully demonstrate the application potential of the multifunctional polymer-metal lattice composites, smart lattice structures with stretching or compressing were developed and tested in this work.

## 2. Materials and Methods

### 2.1. Materials

Potassium permanganate (KMnO_4_, >99.5%), sodium hydroxide (NaOH, >99.5%), hydrochloric acid (HCl, 36.0–38.0%), hydrazine hydrate (N_2_H_4_·H_2_O, >85.0%), sodium chloride (NaCl, >99.5%), nickel sulfate heptahydrate (NiSO_4_·7H_2_O, >99.5%), sodium hypophosphite monohydrate (NaH_2_PO_2_·H_2_O, >99.5%), sodium citrate (Na_3_C_6_H_5_O_7_, >99.5%), ammonia (NH_3_·H_2_O, >25%), copper sulfate pentahydrate (CuSO_4_·5H_2_O, >99.0%), glyoxylic acid monohydrate (CHOCOOH·H_2_O, >99.8%), ethylenediaminetetraacetic acid (EDTA, C_10_H_16_N_2_O_8_, >99.5%), 2,2′-bipyridyl (C_10_H_8_N_2_, >99.0%), potassium ferrocyanide trihydrate (K_4_FeC_6_N_6_·3H_2_O, >99.0%), and potassium hydroxide (KOH, >90.0%) were purchased from Sinopharm Chemical Reagent Co., Ltd., Shanghai, China. The organic passivation solution was purchased from Dongguan Rongxin Coating Technology Co., Ltd., Dongguan, China. Concentrated colloidal palladium Act PP-950 was purchased from Hong Kong Ensen Colloidal Palladium Co., Hong Kong, China. All chemicals were used as received.

### 2.2. VPP 3D Printing

All the samples were designed by Cero 8.0 software (PTC, Boston, MA, USA) and exported in .stl format. Then these files were imported into slicing software (Materialise Magics 21.0, Shanghai, China) for generating G-gode codes recognized by the following printer. The lattice frames were fabricated via an industrial-grade SLA 3D printer (Lite 600HD, UnionTech, Shanghai, China) with a commercial photopolymer (SH8801, UnionTech). The layer thickness, a key print parameter of SLA 3D printing, was set to a minimum of 50 μm for high manufacturing precision. The printed samples were cleaned in isopropanol (IPA) for 1 min (25 °C) and then post-cured with a 365 nm UV light at 60 °C.

### 2.3. Electroless Plating

#### 2.3.1. Pretreatment of ELP

The pretreatment process of electroless plating included: (1) etching, (2) sensitization-activation, and (3) acceleration. The samples were etched in 60 g/L KMnO_4_ and 30 g/L NaOH solution at 50 °C for 1 min to make a rough outer surface. Then, the samples were immersed into a mixture of 75 mL/L HCl and 60 mL/L N_2_H_4_·H_2_O for about 3 h to remove KMnO_4_ residues from polymer surfaces. Thoroughly washed in DI water, the lattice frames were submerged in colloid palladium containing 60 g/L of NaCl, 230 mL/L of HCl, and 10 mL/L concentrated palladium-stannous colloid (Act PP-950) for 10 min at 25 °C. A concentrated palladium-stannous colloid was made of palladium chloride (PdCl_2_), stannous chloride (SnCl_2_), and HCl, which is a solution of complex ions and colloidal particles. After DI water rinsing, the acceleration step was conducted by soaking the samples in 10 vol% HCl at 40 °C for 5 min, which reduced Pd^2+^ to Pd^0^ metallic particles by Sn^2+^ ions and simultaneously removed excess colloid to expose the Pd^0^ catalysts.

#### 2.3.2. Electroless Nickel Plating Process

The lattice frames were immersed in an alkaline electroless nickel plating bath (20 g/L of NiSO_4_·7H_2_O, 30 g/L of NaH_2_PO_2_·H_2_O, and 10 g/L Na_3_C_6_H_5_O_7_) at 38 °C for 15 min to deposit Ni-P layer. The pH value of the bath was set to 8.5 using NH_3_·H_2_O solution.

#### 2.3.3. Electroless Copper Plating Process

The ingredients of the electroless copper plating bath were 12.5 g/L CuSO_4_·5H_2_O as the copper ion source, 15 g/L CHOCOOH·H_2_O as the reducer, 70.1 g/L EDTA as the complexing agent of copper ion, 10 ppm 2,2′-bipyridyl, and 10 ppm K_4_FeC_6_N_6_·3H_2_O as the stabilizer. The pH value of the bath was adjusted to 12.5 using 4 mol/L KOH solution and the plating temperature was kept at 65 °C via water-bath heating.

### 2.4. Characterization

The thickness of the copper layer was measured by an electrolytic metal thickness gauge based on the Faraday electrolysis principle (DJH-G, Wuhan Research Institute of Materials Protection, Wuhan, China).

To measure the hydrophilicity of the resin surface, a drop of deionized water was placed on the sample surface (20 mm × 20 mm × 3 mm) and measured at room temperature (25 °C) using a contact angle tester (Theta Flex, Biolin, Espoo, Finland).

The scanning electron microscope (SEM) imaging and energy dispersive spectroscopy (EDS) mapping were conducted using a field-emission scanning electron microscope (FE-SEM) (Ultra Plus FE-SEM, Carl Zeiss, Oberkochen, Germany) with integrated EDS (X-Max 20, Oxford Instrument, Oxford, UK) for indicating the microstructure and element distribution.

The X-ray photoelectron spectroscopy (XPS) analysis was carried out using an X-ray photoelectron spectrophotometer (PHI 5000 VersaProbe, ULVAC-PHI Inc., Chigasaki, Japan).

The tape test is performed by using a knife to draw grid lines at 1 mm intervals on the surface, then the pressure-sensitive adhesive tape is glued onto the metal coating, and finally, the tape is peeled off. The adhesive strength of the coating can be assessed by examining the percentage of metal film that is removed from the substrate.

### 2.5. Demonstrator Design

**Figure 2:**
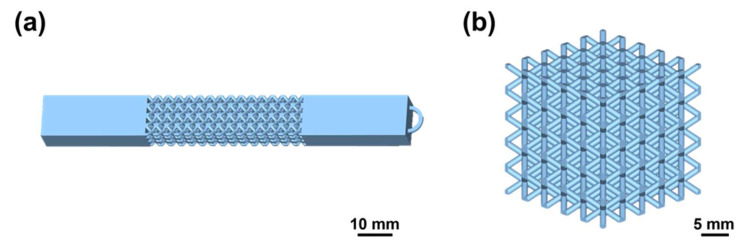
Diagram of the printed lattice structure. The structural design drawings of (**a**) stretching sample and (**b**) compression sample.

Lattice structures with stretching and compression are shown in Figure 2a,b. The dimension of the lattice cell was 3 mm × 3 mm × 3 mm and the strut diameter was set to 0.6 mm. The number of cells of the stretched sample in length, width, and height direction was 4, 3, and 15, respectively (Figure 2a). A stretching sample with clamping parts at both sides was designed. The dimensions of both clamping parts are 30 mm × 9 mm × 12 mm. A small ring at one end was used for the fixture during the plating process. For the compression sample, the cell numbers in three dimensions were 5 × 5 × 5 (Figure 2b) and the cell size was 5 mm × 5 mm × 5 mm.

## 3. Result and Discussion

### 3.1. Quality of VPP 3D Printed Lattice Structures

**Figure 3:**
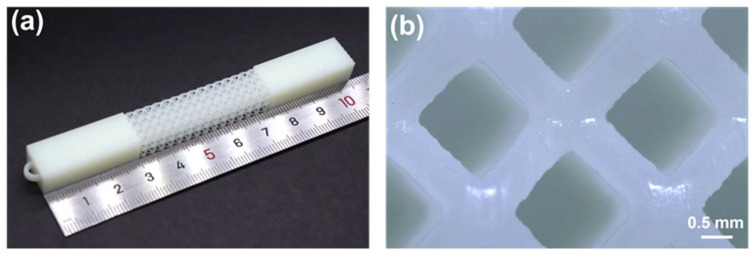
The physical picture of VPP 3D printed samples. (**a**) Photo of overall lattice structure. (**b**) Details of the lattice cell.

After VPP 3D printing, the physical drawing of the sample was shown in Figure 3a,b. Its dimensions were measured under an optical microscope. The average lattice cell size and the rod diameter were measured as 3.09 mm and 0.69 mm, which was close to the design value of cell size (3 mm) and the rod diameter (0.6 mm).

### 3.2. Experiments of Electroless Plating Process

Electroless plating (ELP) is a widely applied wet chemical metallization method [36]. It uses the noble metal palladium (Pd) as a catalyst to activate the redox reaction of metal ions with a strong reducer, thus enabling the electrochemical deposition of highly conductive metal conductors (copper, silver, gold, nickel-phosphorus alloys, etc.) on the surface of the glass, plastic, polymer, ceramic, etc.

#### 3.2.1. Etching

**Figure 4:**
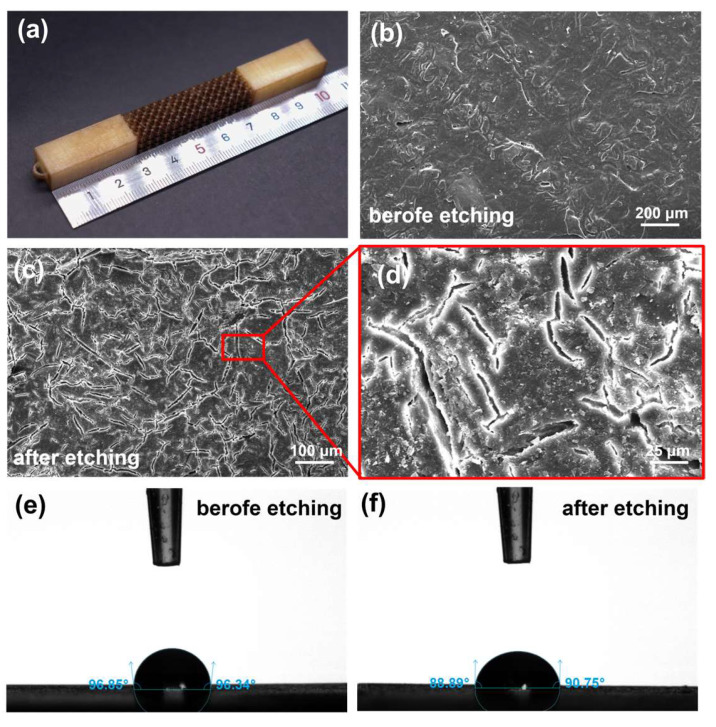
The characterization of the etching step in the electroless plating process. (**a**) The photo of the lattice structure after etching. The surface SEM images of the printed structures (**b**) before and (**c**,**d**) after the KMnO_4_ etching process. Contact angle measurement of a water drop on the surface (**e**) before and (**f**) after etching.

To increase the roughness and hydrophilicity of the polymer surface, a strong oxidizing KMnO_4_ solution was used in the etching step. A large amount of KMnO_4_ remained on the polymer surface, and the sample color turned from white to brown (Figure 4a). Compared to the SEM images before and after etching, plenty of cracks were formed on the polymer surface (Figure 4b–d). Moreover, the contact angle decreased from 96.60° to 89.82° (Figure 4e,f), which indicated that the hydrophilicity of the surface was improved.

#### 3.2.2. Sensitization-Activation and Acceleration

**Figure 5:**
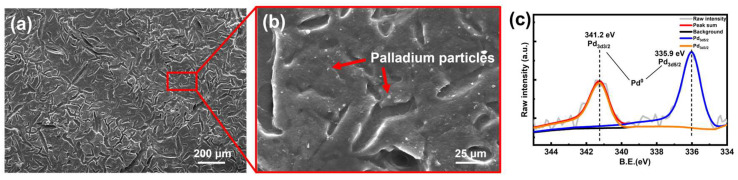
Characterization of palladium catalyzed resin substrate. (**a**,**b**) SEM results of the polymer surface after the acceleration step. (**c**) The XPS result of the Pd element after the acceleration process.

After the etching step, the lattice frame needed to absorb noble metal catalysts to initialize the subsequent ELP reaction. When the etched lattice frames were immersed in the *Pd*/*Sn* catalyst colloid and then accelerated with 10 vol% HCl solution, the following reaction occurred during the acceleration process to produce *Pd*^0^ catalytic particles, which were uniformly attached to the lattice surface (Figure 5a,b).

(1) $${Sn}^{2+}+Pd^{2+}\to {Sn}^{4+}+Pd^{0}$$

As shown in Figure 5b, the metallic palladium particles were uniformly distributed on the polymer surface. Additionally, X-ray photoelectron spectroscopy (XPS) identified the valence states of *Pd* elements (Figure 5c). The spectra were corrected by the C1s peak at 284.8 eV. *Pd* 3d peaks were composed of *Pd* 3d_5/2_ (335.9 eV) and *Pd* 3d_3/2_ (341.2 eV), which corresponded to *Pd*^0^ [37].

#### 3.2.3. Electroless Nickel Plating Process

Alkaline nickel plating was used in our work for the ELP of *Ni*-*P*. The basic chemical reaction is as follows:

(2) $${Ni}^{2+}{+H}_{2}PO_{2}^{-}+3OH^{-}\overset{Pd/Ni}{\to }Ni+HPO_{3}^{2-}+2H_{2}O$$

First, the nickel ions were reduced to nickel by the catalytic action of metallic palladium particles and then the autocatalytic reactions occurred for continuous nickel plating. Simultaneously, a secondary reaction of hypophosphite and hydrogen atoms produced elemental phosphorus.

(3) $$H_{2}PO_{2}^{-}\overset{Pd/Ni}{\to }PO_{2}^{-}+2H$$

(4) $$H_{2}PO_{2}^{-}+H\overset{Pd/Ni}{\to }P{+OH}^{-}+H_{2}O$$

**Figure 6:**
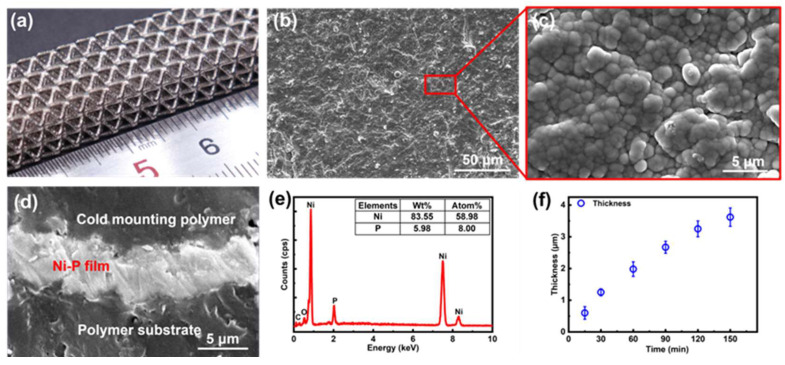
The characterization of the nickel layer. (**a**) The photo of the nickel-coated lattice structure. (**b**,**c**) The SEM images of the nickel layer surface. (**d**) The SEM image of the cross-section of the nickel layer after 150 min ELP. (**e**) The EDS result of the nickel layer. (**f**) The thickness of the nickel layer changing with the electroless plating time.

After ELP, a bright and dense *Ni*-*P* layer could be deposited on the surface of the photopolymer (Figure 6a). The microstructure of the surface and cross-section of *Ni*-*P* film was investigated via SEM imaging (Figure 6b–d). As shown in Figure 6b,c, the *Ni*-*P* layer was dense with no cracks. After 150 min ELP, the thickness of the *Ni*-*P* layer could reach around 3.7 μm (Figure 6d,f), and the resistivity of the nickel layer was approximately $8.2\times 10^{-7}\Omega \cdot m$ . EDS investigation illustrated that the concentration of phosphorus and nickel in the alloy film was 5.98 wt% and 83.55 wt%, respectively (Figure 6e).

#### 3.2.4. Electroless Copper Plating Process

For the ELP of copper, *Pd*^0^ plating seeds were also the catalyst of the following chemical reaction:

(5) $$Cu{\left(EDTA\right)}^{2-}+2CHOCOOH+4OH^{-}\overset{Pd^{0}/Cu^{0}}{\to }Cu+EDTA^{4-}+2HC_{2}O_{4}^{-}+2H_{2}O+H_{2}↑$$

**Figure 7:**
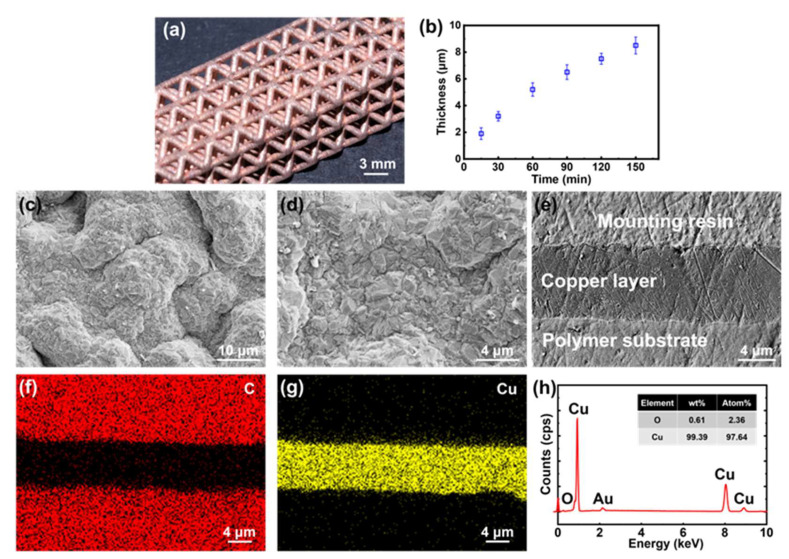
The characterization of the copper layer. (**a**) The photo of the copper-coated lattice structure. (**b**) The thickness of the copper layer changes with the electroless plating time. (**c**,**d**) The SEM images of the copper layer surface. (**e**) The SEM image of the cross-section of the copper layer after 150 min ELP. Elemental mapping of (**f**) C and (**g**) Cu in the cross-section. (**h**) The EDS result of the nickel layer.

Then, the copper element could be continuously deposited around the *Cu*^0^ particles to form a series of copper spheres. These spheres grew larger and then connected as a dense copper layer (Figure 7a,d). As shown in Figure 7b,c,e, the thickness of the copper layer could reach 8 μm after 150 min ELP. The resistivity of the copper layer calculated according to Pouillet’s law was around $2.0\;\times 10^{-8}\;\Omega \cdot m$ , which was close to the pure copper ( $1.7\;\times 10^{-8}\;\Omega \cdot m$ ). The low resistivity of the copper layer was due to the consistency and high purity of the copper layer. As shown in SEM images, the deposited copper layer was dense without any cracks or pores (Figure 7c–g). The EDS result showed that the purity of the copper layer was higher than 99 wt% (Figure 7h).

### 3.3. Characterization

#### 3.3.1. Mechanical Strength

**Figure 8:**
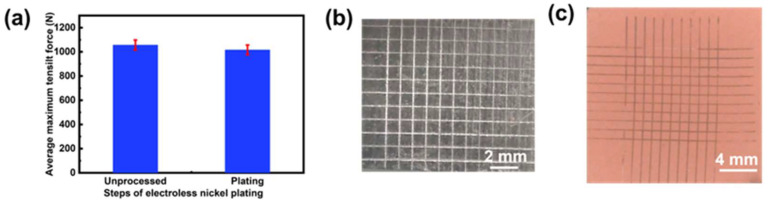
ELP characterization. (**a**) The tensile strength of lattice samples before and after the ELP process. (**b**) Nickel layer after the tape test. (**c**) Copper layer after the tape test.

Lattice stretching samples were prepared as described in Section 2.3 in order to investigate the effects of the ELP step on the mechanical strength. The average maximum tensile force decreased slightly from 1056 N to 1016 N by only 3.79% (Figure 8a), which meant that the ELP step had almost no effect on the mechanical properties of the lattice structures.

#### 3.3.2. Adhesion Strength

In order to test the adhesion strength of the ELP nickel and copper metal layers on the polymer, tape tests, according to ASTM D3359-09 [38], were conducted on the clamping parts of the sample for a flat substrate to draw cut grids. The result showed that no metal layer was peeled off, which indicated the best adhesion grade of 5B (Figure 8b,c). The adhesion strength of the lattice part could be approximately evaluated by this result since the whole part underwent the same ELP procedures.

### 3.4. Demonstrator

**Figure 9:**
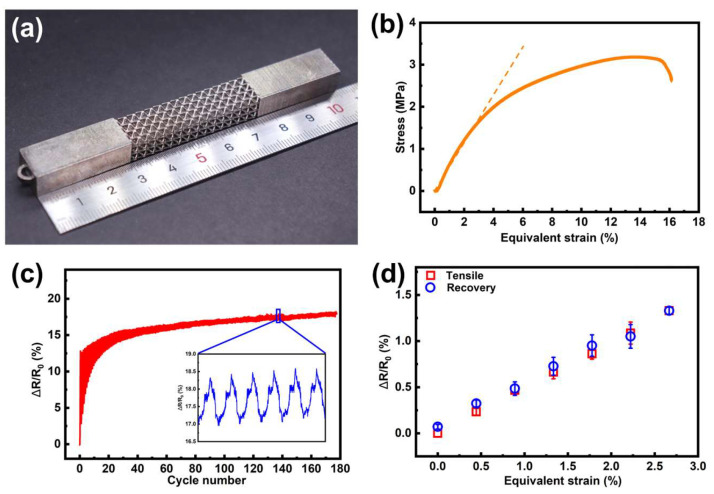
The stretching self-sensing test results. (**a**) The photo of the stretching lattice structure. (**b**) Stress-equivalent strain curve of the stretching lattice (The slope of the dot line is the elastic modulus). (**c**) $\Delta R/R_{0}$ of the lattice with 3% applied strain during about 180 stretching–releasing cycles. (**d**) The $\Delta R/R_{0}$ curve at different strains.

**Figure 10:**
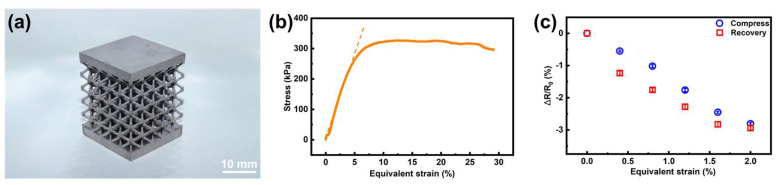
The compression self-sensing test results. (**a**) The photo of the compression lattice structure. (**b**) Stress-equivalent strain curve of the compression lattice (The slope of the dot line is the elastic modulus). (**c**) $\Delta R/R_{0}$ the curve at different strains.

As described in Section 3.2.3 and Section 3.2.4, Ni-P film has a much higher resistivity than its copper counterpart. This was quite preferable for resistive sensors since the induced larger original resistance could provide more obvious resistance variation, making the signal easy to detect. Moreover, Ni-P alloy has better oxidation and corrosion resistance compared to pure copper. Therefore, the smart lattice structures with tensile and compressive strain self-sensing functionality were developed with conformal Ni-P film strain gauges, respectively (Figure 9a and Figure 10a).

The resistance response of the sensor was acquired in real time from the Wheatstone bridge circuit. No significant delay in sensor response was observed for the entire experimental device. The tensile or compressive equivalent strain $\varepsilon_{e}\;$ and the stress $\sigma_{s}$ is calculated using the following formulas:

(6) $$\varepsilon_{e}=\frac{\Delta l}{l_{0}}$$

(7) $$\sigma_{s}=\frac{F}{A}$$

Δ*l* represents the deformation variable for tensile or compression of the lattice structure and *l*_0_ represents the initial length or height of the lattice structure. *F* is the uniaxial reaction force caused by the input loading and *A* is the cross-sectional area. The sensitivity of the strain sensor is evaluated using gauge factor (GF), which can be expressed using the following equation:

(8) $$G=\frac{\Delta R/R_{0}}{\Delta l/l_{0}}$$

where $\Delta R/R_{0}$ represents the normalized relative resistance change ( $\Delta R=R_{i}-R_{0}$ , *R_i_* and *R*_0_ mean the resistance under applied strain and the initial resistance, respectively).

For the smart lattice structures with tensile strain self-sensing functionality, a fracture tensile test was first conducted to reveal the elastic deformation range. A typical stress-equivalent strain curve was shown in Figure 9b. A near-linear tensile behavior exhibited at the strain range of 0–3%; as such, this range could be regarded as the elastic range of the lattice sample. The following tests were conducted within this range to ensure the lattice structure returned to its original state. According to previous works about strain sensors, a large fluctuation of resistance output occurred in the very first few cycles of the tensile experiment [39,40]. This behavior might be related to the competition between disruption and reconstruction of the conductive network. Therefore, test samples were first stretched in cycles for fatigue aging. Figure 9c showed the resistance output for about 180 cycles with a strain range of 0–3% and a stretching rate of 1 mm/min. The repeatability and reliability of the sensor output were largely improved after 80-cycle aging, which showed a desirable durability of the self-sensing lattice for long-term applications.

For the static test, five samples were first aged for 150 cycles, and then tensile tested. According to Figure 9d, with the gradual increase of the equivalent strain from 0 to 2.7%, the output ΔR/R_0_ increased linearly to around 1.3%. When the tensile loading was released step by step, the output could return to zero point. The tensile and recovery data were nearly overlapped, indicating that the hysteretic behavior was not remarkable. In cyclic testing of lattice structures, the initial instability of strain change amplitude can be attributed to the following factors. There are micro-cracks caused in the AM or ELP coating process, which might propagate initially, causing a sharp strain change at the beginning. As the material undergoes more cycles, it tends to settle into a more stable deformation pattern. The GF of the tensile-sensing lattice was about 0.50, calculated by Equation (8).

For the compression self-sensing test, to demonstrate the response of the sensor to external compressions, the smart lattice structure (Figure 10a) was secured between two cylinders of the universal testing machine. The lattice was compressed to the densification stage and, similarly, stress-equivalent strain curves were also attained (Figure 10b). The compression lattice exhibited the elastic behavior at the strain range of 0–5%. Therefore, the compression experiments were performed in the strain range of 0–2% with 0.4% strain intervals. According to Figure 10c, the resistance of the sensor reduced with the increasing strain due to the compression of the Ni-P layer. After that, moving the upper cylinder to its initial position step-by-step, the resistance almost returned to its initial value. The GF of the compression-sensing lattice structure was calculated to be 1.44.

The resistance of a regular conductor can be expressed as:

(9) $$R=\rho \frac{L}{A}$$

where ρ (Ω·cm) is the resistivity, *L* (cm) is the length of the conductor, and *A* (cm^2^) is the cross-section area of the conductor. When the lattice structure is subjected to tensile or compressive loads, the lattice rods experience the corresponding tensile or compressive strains. Consequently, there are dimensional changes in the Ni-P coating layer, affecting its resistance. Given the thin layer thickness of the small Poisson’s ratio of the metal coating, the variations in the coating area can be disregarded. The tensile or compressive strain primarily influences the length of the coating conductor. Therefore, the resistance of the coating increased with tensile strain, while decreased with compressive strain.

## 4. Conclusions

In this work, we proposed a novel HAM method that integrates VPP 3D printing and ELP metallization processes to fabricate multifunctional lattice structures. With VPP 3D printing, a highly complex photopolymer lattice framework was obtained and then pretreated for attaching the metallic palladium particles as the ELP catalysts. After 150 min of ELP, the resistivity of the Ni-P and copper films could reach $8.2\times 10^{-7}\Omega \cdot m$ and $2.0\;\times 10^{-8}\;\Omega \cdot m$ , respectively. The ELP step had no negative effect on the mechanical properties of the polymer-metal composite lattice structure and the adhesion between the metal and the resin was 5B, the highest grade of ASTM standard. Based on the proposed HAM technology, smart lattice structures with self-sensing functionality for both compressive and tensile loads were successfully developed to verify the application potential of this HAM method. For the future works, the adhesion durability of the copper layer on the lattice structure, the repeatability of the sensor signals, and the long-term working stability of the smart lattice structure will be further investigated to promote the practical application of this technology.

## Data Availability

Data are contained within the article.
